# A cross-sectional study: comparison of public perceptions of adverse drug reaction reporting and monitoring in eastern and western China

**DOI:** 10.1186/s12913-022-07720-0

**Published:** 2022-03-08

**Authors:** Ningsheng Wang, Yue Chen, Biqi Ren, Yufang Xiang, Nan Zhao, Xianyan Zhan, Bianling Feng

**Affiliations:** 1 The Department of Pharmacy Administration and Clinical Pharmacy, School of Pharmacy, Xi’an Jiaotong University, Xi’an, Shannxi, China; 2 The School of Public Health, Xi’an Jiaotong University, Xi’an, Shannxi, China; 3 The First Affiliated Hospital of Xi’an Jiaotong University, Xi’an, Shaanxi, China

**Keywords:** Adverse drug reaction, Pharmacovigilance, Knowledge, attitudes and practice, General public

## Abstract

**Background:**

Adverse drug reactions (ADRs) cause a substantial clinical and economic burden. Spontaneous reporting of ADRs by the public is crucial. In some developed countries like the United States, Canada, consumers have been allowed to directly report ADRs, however, convenient channels for direct ADR reporting by the public are lacking in China.

**Objective:**

We aimed to compare the knowledge, attitudes, and practice(KAP) regarding monitoring and reporting of adverse drug reaction (ADR) among the general public in eastern and western China.

**Methods:**

A questionnaire-guided cross-sectional study was administered to participants in Nanjing and Xi’an during April–July 2019. A descriptive statistical analysis was used to describe respondents’ demographic information and other results. The t-test and analysis of variance were used to test the differences in knowledge and attitudes among respondents with different demographic characteristics. *P* < 0.05 was considered statistically significant. Binary logistic regression analysis was used to examine factors associated with knowledge and attitudes.

**Results:**

A total of 1085 questionnaires were distributed in this survey, 869 valid questionnaires were returned, the recovery rate was 80.09%. Respondents showed poor knowledge of the definition of ADRs and reporting criteria, with a significant difference in average knowledge scores according to education level, gender, and age group. Most respondents had positive attitudes toward ADR monitoring and reporting, with no significant differences in knowledge and attitude scores between the two cities. In total, 68.93% of respondents said they would feedback information to health care professionals, most (84.35%) would take the initiative to report ADRs if there were a convenient method. More than half (58.57%) of respondents were more likely to report ADRs by telephone.

**Conclusion:**

The findings of our study indicated that health care professionals should encourage patients to actively report ADR. China should also explore ways to facilitate direct public reporting of ADRs by improving relevant laws and regulations.

**Supplementary Information:**

The online version contains supplementary material available at 10.1186/s12913-022-07720-0.

## Introduction

The World Health Organization defines “adverse drug reaction” (ADR) as any response to a drug, which is noxious, unintended, and occurs at doses normally used in man for prophylaxis, diagnosis, or treatment of disease [1]. According to previous studies, ADRs are an important cause of morbidity and mortality in all age groups [2, 3]. A review study [4] pointed out that the incidence of ADRs fluctuates between 4.6% and 17.6% in all hospitalizations, and nearly 80% of medical expenses arise from ADRs, ADRs also seriously affect the quality of life of patients. The role of pharmacovigilance is to detect, evaluate, understand, and prevent adverse reactions or any other drug-related problems, and to improve drug safety in patients [5]. The key to reducing the consequences of ADRs and strengthening pharmacovigilance is to identify and promptly report ADRs [6, 7].

The spontaneous reporting system has been the backbone of pharmacovigilance since its introduction in the 1960s, and most countries have an established spontaneous drug reaction reporting system [8–10]. However, underreporting of ADRs is still a widespread problem in spontaneous reporting systems [7, 11, 12]. Therefore, countries encourage and require responsible stakeholders, including health care professionals (HCPs), regulatory authorities, and industry, as well as the public (consumers, patients) to participate in the spontaneous reporting system, for extensive reporting of ADRs [4, 7, 13]. The critical role of patients’ participation in pharmacovigilance and direct reporting of ADRs has been increasingly recognized [8, 14–17]. According to several studies [8, 18–20], reporting of ADRs by patients can increase the number of ADR reports and the rate of ADR reporting; more importantly, patient ADR reporting has many advantages over-reporting by HCPs. For example, patients can provide first-hand, detailed information about ADRs and the environmental factors related to ADR occurrence, which can help to better understand the patient’s experience of ADR, the personal impact on daily life caused by ADR, as well as other ADR-related information [21–25]. Studies [16, 26–28] have also shown that patients are more likely to identify and report new adverse reactions, leading to the improvement of safety signals. ADRs reported by the public can complement those reported by HCPs, to add value to the pharmacovigilance system and generate new safety signals [6, 18, 19]. Globally, more than 40 countries have incorporated patient or consumer ADR reporting into their pharmacovigilance schemes [29, 30]. In some countries like the United States, Canada, and New Zealand, consumers have been allowed to directly report ADRs since implementation of the pharmacovigilance plan [14, 31]. The current reporting channels for ADRs in China are through drug manufacturers, drug trading enterprises, and medical institutions actively collecting and reporting ADRs to the local ADR monitoring centre. While in China, the public has not received sufficient attention as an important group for reporting ADRs. According to the 2019 National Annual Report on the Monitoring of ADRs issued by China [32], reports from the public accounted for only 0.1% of the total. Chinese laws and regulations encourage the public to report ADRs; however, it is generally via an indirect reporting method with patients reporting to HCPs, drug manufacturers, drug trading enterprises, and local ADR monitoring centre [33], there is no convenient way for the Chinese public to directly report ADRs [34, 35].

As early as the “Erik Declaration” of the 1997 Pharmacovigilance Conference, the importance of patients directly participating in pharmacovigilance for ADR reporting has been emphasized [15]. In the context of increasingly more countries allowing the public report of ADRs [7, 8], China should also actively explore ways for the public to report ADRs. Compared with foreign studies, research on ADR monitoring and reporting by the public remains scarce in China. In a previous study [36], we assessed public knowledge, attitudes, and practice(KAP) regarding pharmacovigilance in western China, however, there is no study to compare the public KAP regarding pharmacovigilance in two regions with large economic gap. The purpose of this study was to understand knowledge, attitudes, and practices among the general public in eastern and western China regarding ADR monitoring and the ADR reporting system and compare the KAP among the general public in eastern and western China. Our research results will provide reference and a basis for relevant departments to improve the ADR reporting system in the future in China.

## Methods

### Study area and study population

This questionnaire survey was conducted for 4 months, from April 2019 to July 2019 in Nanjing and Xi’an. China is a developing country with a large population and vast territory, and there are differences in development between the eastern and western parts of the country. According to the China Statistical Yearbook 2019 [37], the regional GDP of the eastern, central, and western regions in 2018 accounted for 52.6%, 21.1%, and 20.1% of the total for all of China, respectively. Therefore, we selected the eastern and western regions as the research areas for comparison and selected one city in the east and one in the west. Nanjing is the capital city of Jiangsu Province in the eastern region, and Xi’an is the capital city of Shaanxi Province in the western region. In 2018, the GDP of Jiangsu Province ranked second among the 11 provinces in the eastern region, and that of Shaanxi Province ranked second among the 12 provinces in the western region [37]. This survey included members of the general public as study participants. To ensure the availability of data, we set the following restrictions for survey respondents: at least 16 years old (General Provisions of Chinese Civil Law stipulates that any citizen who has reached the age of 16 years and whose main source of income is his labor shall be regarded as a person with full capacity for civil conduct [38]) and a permanent resident of the selected cities; no diseases affecting reading, comprehension, or expression; no professional medical or pharmaceutical background; and not engaged in medically related work. We used simple random sampling to select the sample and selected the survey area according to the administrative division of the two cities (each administrative area has 11 administrative areas). We randomly chose three survey locations (subway/bus station, park/scenic area, shopping mall/supermarket) in each administrative area, considering high-traffic areas but excluding tourist areas.

Using the sample size calculation formula (Eq. 1), the final calculated sample size in both cities was *N* = 385; further considering the questionnaire response rate, the sample size was increased to 481 in each of the two cities.

1 $$\mathrm{N}=\frac{{\mathrm{Z}}^{2}\times \mathrm{P}\times (1-\mathrm{P})}{{\upsigma }^{2}}$$

where Z = 1.96, *P* = 0.5 (maximum sample size), and σ = 0.05 (permissible error is 5%).

### Questionnaire design

We conducted a KAP (knowledge, attitude, and practice) assessment of respondents using a questionnaire. The questionnaire was developed based on a review of the published literature, and we further improved the questionnaire design with the advice of experts and a pilot study (including 40 people).

The questionnaire consists of four parts (supplementary file): the first part queried demographic information of respondents. The second part included 10 questions; responses were used to evaluate respondents’ knowledge of ADR monitoring and the reporting system, including the definition of ADR, responsibilities of the ADR monitoring centre and its regulatory scope. Responses scored 1 point for each correct answer and 0 points for each wrong answer. The third section included eight questions addressing respondents' attitudes toward the ADR monitoring system, such as the importance of collecting ADR information. A five-point Likert scale (strongly agree = 5, agree = 4, unsure = 3, disagree = 2, and strongly disagree = 1) was used to evaluate questions about the attitudes of respondents. For statistical analysis, we divided response options for questions about attitude into two categories: positive and negative attitudes. Responses indicating a positive attitude included “strongly agree” and “agree”; negative attitude included “unsure”, “disagree” and “strongly disagree”. The fourth part of the questionnaire assessed respondents’ practice included seven questions: Q1. Would you consult doctors or pharmacists for ADR information while purchasing drugs?Q2. Would you check the "adverse drug reactions" section of the drug instructions?Q3. Do you suspect that ADR is occurring when you feeling sick?Q4. What measures would you take when you have an adverse drug reaction?Q5. If there is a policy that makes it easier for patients to report adverse drug reactions, would you take the initiative to report?Q6. Which way do you prefer to report adverse drug reactions? Q7. Why do you think you did not report adverse drug reactions?

### Data collection and data analysis

The survey was conducted by way of on-site questionnaire distribution and on-site recycling. An informed consent statement was attached to the first page of the questionnaire, to explain the purpose of the survey. The content of the questionnaire did not involve the personal information of respondents, to ensure anonymity and confidentiality of responses. After excluding invalid questionnaires, we used EpiData version 3.1 for double-entry of data from all valid questionnaires, to ensure the quality of the data. Cleaned data were exported to IBM SPSS version 24.0 for analysis (IBM Corp., Inc., Armonk, NY, USA). We used descriptive statistical analysis, such as frequency and percentage, to describe respondents’ demographic information and other results. The t-test, and analysis of variance were used to test the differences in knowledge and attitudes scores among respondents with different demographic characteristics. The difference test of knowledge and attitude scores of respondents of different genders and ethnicities uses t-test, and the difference test of knowledge and attitude scores of respondents of different ages, education levels, and annual income uses analysis of variance. In order to understand the factors associated with public knowledge and attitudes, the average score of public knowledge was converted into 2 dichotomies: adequate knowledge and inadequate, and a cut-off point was established to categorize knowledge score, in the same way, convert the average score of public attitudes into 2 dichotomies: positive attitudes and non-positive attitudes, and a cut-off point was established to categorize attitude scores. We considered setting the average score of knowledge and attitude as the cut-off point respectively. The public’s knowledge score was greater than or equal to the average score: sufficient knowledge, less than the average score: insufficient knowledge. The attitude score was set in the same way. Binary logistic regression analysis was used to examine factors (age, gender, education, annual income) associated with adequate knowledge and positive attitudes. A level of *P* < 0.05 was accepted as statistically significant. The odds ratios (OR) and their 95% confidence intervals (CI) were calculated in order to estimate relative risk. *P* < 0.05 was considered statistically significant.

## Ethical clearance

This study was conducted after ethical clearance obtained from the medicine biomedical ethics committee of Xi’an Jiaotong University, Xi’an, Shannxi, China[NO.2019–1233]. Information about the objective and contents of the study, as well as their right to refuse before any data collection, was given to all participants. Written informed consent was obtained from all individual participants included in the study. As human participants are 16 years old, we confirm informed consent was obtained from all subjects their legal guardian(s). All methods were carried out in accordance with relevant guidelines and regulations.

## Results

### Demographic information

A total of 1085 questionnaires were distributed in this survey (Nanjing:563, Xi’an:522). After collecting completed questionnaires, we excluded incomplete questionnaires, illogical questionnaires, and questionnaires answered by respondents with medical-related education/professional backgrounds. Finally, a total of 869 valid questionnaires were returned (Nanjing:433, Xi’an:436), the recovery rate was 80.09%. Among the 869 valid questionnaires, there were more male than female respondents (457 and 402, respectively). The largest proportion of respondents was those age 18–30 years (59.26%), differences in the distribution of respondents by age, education level, and income level between the two cities were significant, other demographic information was shown in Table 1. Table 2 shown that in Nanjing and Xi’an, among respondents age over 51 years, 31.82% and 26.92%, respectively, had lower education levels (junior high school or below). Among respondents under age 50 years, 8.74% and 6.70%, respectively, had lower education levels. In other words, there were more respondents in older age groups with lower education levels in both cities.

**Table 1:** Demographic information, China, 2019

Characteristic	Total	Nanjing	Xi’an	*P*
	n(%)	n(%)	n(%)	
**Gender**				
Male	457(52.59)	241(55.66)	216(49.50)	0.095
Female	406(46.72)	191(44.11)	215(49.30)	
Unfilled	6(0.69)	1(0.23)	5(1.10)	
**Age**				
< 18	26(2.99)	21(4.85)	5(1.10)	0.017*
18–30	515(59.26)	260(60.05)	255(58.50)	
31–40	141(16.23)	70(16.17)	71(16.30)	
41–50	84(9.67)	38(8.78)	46(10.60)	
51–60	49(5.64)	18(4.15)	31(7.10)	
61–70	36(4.14)	21(4.85)	15(3.40)	
> 70	12(1.38)	5(1.15)	7(1.60)	
Unfilled	6(0.69)	0(0.00)	6(1.40)	
**Ethnicity**				
Han	850(97.81)	423(97.69)	427(97.90)	0.052
Other	13(1.50)	10(2.31)	3(0.70)	
Unfilled	6(0.69)	0(0.00)	6(1.40)	
**Education level**				
None/primary	17(1.96)	8(1.85)	9(2.10)	0.044*
Junior school	70(8.06)	40(9.24)	30(6.90)	
High school	188(21.63)	92(21.25)	96(22.00)	
Junior college	188(21.63)	76(17.55)	112(25.70)	
Undergraduate college	352(40.51)	192(44.34)	160(36.70)	
Master degree/above	49(5.64)	25(5.77)	24(5.50)	
Unfilled	5(0.58)	0(0.00)	5(1.10)	
**Annual income level**				
Under ¥30,000	382(43.96)	199(45.96)	183(42.00)	0.014*
¥30,000-¥60,000	194(22.32)	84(19.40)	110(25.20)	
¥60,000-¥90,000	145(16.69)	65(15.01)	80(18.30)	
¥90,000-¥120,000	72(8.29)	37(8.55)	35(8.00)	
¥120,000-¥150,000	41(4.72)	22(5.08)	19(4.40)	
Above ¥150,000	35(4.03)	26(6.00)	9(2.10)	

*Significant difference with *p* value < 0.05

**Table 2:** Relationship between education level and age, China, 2019

			**Education level**	
			Junior high school and below n (%)	High school and above n (%)
**Age**	Nanjing	< 50	34(8.74)	355(91.26)
	Xi’an		25(6.70)	348(93.30)
	Nanjing	> 51	14(31.82)	30(68.18)
	Xi’an		14(26.92)	38(73.08)

### Knowledge levels regarding ADRs

In knowledge section, the question with the lowest rate of correct answers (31.42%) was regarding the criteria for reporting ADR (If an adverse reaction is suspected, report it immediately). Question on the definition of ADR also had low rates of correct answers, with only about half of respondents choosing the correct answer (51.90% and 47.41% for Questions 1 and 2, respectively). The average knowledge score was 6.49 points, with 6.42 and 6.56 in Nanjing and Xi’an, respectively, there was no significant difference in knowledge scores between the two cities (*P* = 0.096). As shown in Table 3, differences in the average knowledge scores among respondents in Nanjing with different education levels were significant among them, the group with a junior high school education level had the lowest average score per person (5.83). In pairwise comparison, the difference between average scores of respondents with a junior high school education and those with a technical secondary school, high school, or university level education was statistically significant. In Xi’an, differences in average knowledge scores by gender and age groups were significant, the average knowledge score among female participants (6.73) was higher than that of male respondents (6.37). In different age groups, the lowest average score (5.27) was among respondents age 61–70 years, with differences between this age group and respondents age less than 18 years, 18–30 years, 31–40 years, and 41–50 years.

**Table 3:** Knowledge scores of respondents, China, 2019

Characteristic	Nanjing		Xi’an	
	**Mean(SD)**	***P*-value**	**Mean(SD)**	***P*-value**
**Gender**				
Male	6.51 (1.53)	0.196	6.37 (1.74)	0.027*
Female	6.31 (1.44)		6.73 (1.67)	
**Age**				
< 18	5.90 (1.84)	0.787	7.20 (1.92)	0.014*
18–30	6.45 (1.50)		6.65 (1.61)	
31–40	6.39 (1.54)		6.62 (1.92)	
41–50	6.50 (1.13)		6.54 (1.73)	
51–60	6.33 (1.28)		5.84 (1.62)	
61–70	6.48 (1.63)		5.27 (1.75)	
> 70	6.80 (1.79)		6.86 (1.77)	
**Ethnicity**				
Han	6.41 (1.50)	0.215	6.54 (1.72)	0.428
Other	7.00 (0.94)		7.33 (0.58)	
**Education level**				
None/primary	7.38 (1.92)	0.036*	5.00 (1.82)	0.078
Junior school	5.83 (1.68)		6.23 (1.76)	
High school	6.42 (1.45)		6.44 (1.76)	
Junior college	6.28 (1.44)		6.66 (1.62)	
Undergraduate college	6.54 (1.46)		6.65 (1.69)	
Master degree/above	6.56 (1.42)		6.63 (1.76)	
**Annual income level**				
Under ¥30,000	6.39 (1.60)	0.291	6.54 (1.66)	0.601
¥30,000-¥60,000	6.19 (1.41)		6.44 (1.78)	
¥60,000-¥90,000	6.66 (1.50)		6.75 (1.59)	
¥90,000-¥120,000	6.57 (1.24)		6.83 (1.50)	
¥120,000-¥150,000	6.41 (1.40)		6.37 (2.48)	
Above ¥150,000	6.58 (1.24)		6.00 (1.87)	

*Significant difference with *p* value < 0.05; *SD* Std. Deviation

### Respondents’ attitudes regarding ADRs

In general, respondents had a positive attitude toward the ADR monitoring system, the number of people who kept positive attitude to each question exceeds 85.0% of the total. The highest proportion of positive responses (96.89%) was for the question “Doctors and pharmacists should inform consumers (patients) about ADRs”, followed by 95.05% of respondents giving a positive response to the question “Collection of ADR information can improve the safety of drug use”. The average attitude score among all respondents was 4.38, and that of Nanjing and Xi’an was 4.38 and 4.37, respectively, there was also no significant differences in attitude scores between the two cities (*P* = 0.928). As shown in Table 4, in Nanjing, the difference in average scores by age group was significant, and the lowest average attitude score was among respondents younger than 18 years old, which was significantly different in comparison with other the five age groups. In Xi’an, average scores according to educational level were significantly different. Differences in average scores of respondents with a junior high education and those with a bachelor’s degree and master's degree or above were significant. In both Nanjing and Xi’an, the difference in per capita score among different income groups was statistically significant. Binary logistic regression analyses the influencing factors related to the knowledge and attitudes of the public in Nanjing and Xi’an. No independent influencing factors of public knowledge were found in Nanjing, while logistic regression analysis of Xi’an showed that gender was an independent influencing factor of knowledge, and female knowledge was more adequate (*P* = 0.005, OR = 1.930, 95%CI: 1.219–3.056). Age is an independent influencing factor of public attitude in Nanjing. With the increase of age, the public is more likely to have a positive attitude (*P* = 0.015, OR = 1.033, 95%CI: 1.006–1.061), no independent influencing factor related to the public attitudes in Xi’an was found.

**Table 4:** Attitude scores of respondents, China, 2019

Characteristic	Nanjing		Xi’an	
	**Mean(SD)**	***P*-value**	**Mean(SD)**	***P*-value**
**Gender**				
Male	4.37 (0.49)	0.844	4.38 (0.47)	0.857
Female	4.38 (0.44)		4.37 (0.46)	
**Age**				
< 18	4.04 (0.60)	0.023*	4.10 (0.21)	0.116
18–30	4.36 (0.46)		4.38 (0.46)	
31–40	4.44 (0.41)		4.47 (0.50)	
41–50	4.38 (0.53)		4.30 (0.46)	
51–60	4.58 (0.44)		4.33 (0.44)	
61–70	4.52 (0.50)		4.17 (0.49)	
> 70	4.38 (0.29)		4.55 (0.41)	
**Ethnicity**				
Han	4.38 (0.47)	0.494	4.37 (0.47)	0.749
Other	4.28 (0.41)		4.46 (0.51)	
**Education level**				
None/primary	4.13 (0.38)	0.239	4.22 (0.65)	0.033*
Junior school	4.43 (0.51)		4.18 (0.44)	
High school	4.29 (0.47)		4.33 (0.45)	
Junior college	4.41 (0.48)		4.35 (0.47)	
Undergraduate college	4.40 (0.45)		4.45 (0.46)	
Master degree/above	4.37 (0.52)		4.45 (0.45)	
**Annual income level**				
Under ¥30,000	4.33 (0.47)	0.039*	4.31 (0.45)	0.001*
¥30,000-¥60,000	4.44 (0.49)		4.38 (0.49)	
¥60,000-¥90,000	4.30 (0.41)		4.41 (0.47)	
¥90,000-¥120,000	4.39 (0.41)		4.46 (0.38)	
¥120,000-¥150,000	4.56 (0.45)		4.55 (0.45)	
Above ¥150,000	4.54 (0.58)		4.51 (0.51)	

*Significant difference with *p* value < 0.05; *SD* Std. Deviation

### Practices regarding ADRs

More than half of respondents (67.20%) stated they consult a doctor or pharmacist for information about ADRs before using a drug (Q1). If an ADR occurred, most people (68.93%) said they would feedback the information to an HCP (Q4). When asked if they would report an ADR (Q5), most respondents (84.35%) said they would take the initiative to report if there were a policy to allow patients to more easily report ADRs. Regarding the reporting method (Q6), more than half (58.57%) of respondents said they preferred to report an ADR by telephone, and about one-third said they would rather complete an online form (28.88%). As far reasons for not reporting ADRs (Q7), 56.39% and 44.88% of the total responded “I do not know where to feedback ADR information” and “I think that ADRs are not very serious and it is not necessary to report an ADR”, respectively. Regarding the reason for not reporting an ADR, responses were significantly different between participants in the two cities (Q7). Compared with respondents in Xi’an, a higher proportion of those in Nanjing chose the responses “I don’t want to stop discontinue medication because of an ADR” and “I think it is meaningless to report ADRs and don’t want to report an ADR” (Table 5).

**Table 5:** Practices regarding ADRs among respondents, China, 2019

Questions/answers	**Nanjing**		**Xi’an**		**Total**	
	Number (n)	Frequency (%)	Number (n)	Frequency (%)	Number (n)	Frequency (%)
**1.Would you consult doctors or pharmacists for ADR information while purchasing drugs?**						
Yes, I would	284(65.59)		300(68.81)		584(67.20)	
No, I wouldn`t	149(34.41)		135(30.96)		284(32.68)	
unfilled	0(0.00)		1(0.23)		1(0.12)	
	*P* = 0.289					
**2.Would you check the "adverse drug reactions" section of the drug instructions?**						
Every time	172(39.72)		174(39.91)		346(39.82)	
Most time	137(31.64)		151(34.63)		288(33.14)	
Sometimes	110(25.41)		101(23.17)		211(24.28)	
Never	14(3.23)		8(1.83)		22(2.53)	
unfilled	0(0.00)		2(0.46)		2(0.23)	
	*P* = 0.438					
**3.Do you suspect that ADR is occurring when you feeling sick?**						
Suspect and check the instructions for more information	262(60.51)		242(55.50)		504(58.00)	
Suspect but not check the instructions for more information	129(29.79)		143(32.80)		272(31.30)	
Not suspect	42(9.70)		49(11.24)		91(10.47)	
unfilled	0(0.00)		2(0.46)		2(0.23)	
	*P* = 0.359					
**4. What measures would you take when you have an adverse drug reaction?**						
Feed the information back to the medical staff	303(69.98)		296(67.89)		599(68.93)	
Nothing was done	69(15.94)		78(17.89)		147(16.92)	
Report to Pharmaceutical trading enterprises (drug store)	46(10.62)		29(6.65)		75(8.63)	
Report to ADR monitoring center	5(1.15)		8(1.83)		13(1.50)	
Report to pharmaceutical Manufacturing companies	2(0.46)		7(1.61)		9(1.04)	
Exposure to the News Media	8(1.85)		6(1.38)		14(1.61)	
unfilled	0(0.00)		12(2.75)		12(1.38)	
	*P* = 0.148					
**5. If there is a policy that makes it easier for patients to report adverse drug reactions, would you take the initiative to report?**						
Yes, I would	362(83.60)		371(85.09)		733(84.35)	
No, I wouldn`t	71(16.40)		62(14.22)		133(15.30)	
unfilled	0(0.00)		3(0.69)		3(0.35)	
	*P* = 0.396					
**6. Which way do you prefer to report adverse drug reactions?**						
By telephone	262(60.51)		247(56.65)		509(58.57)	
By internet	123(28.41)		128(29.36)		251(28.88)	
By email	32(7.39)		35(8.03)		67(7.71)	
By post	16(3.69)		8(1.83)		24(2.76)	
unfilled	0(0.00)		18(4.13)		18(2.07)	
	*P* = 0.380					
**7. Why do you think you did not report adverse drug reactions? (multiple choices)**						
Do not know where to feedback ADR information	238(32.69)		252(36.47)		490(34.53)	
ADR is not too serious to report	200(27.47)		190(27.50)		390(27.48)	
Think it's too much trouble to report the ADR	117(16.07)		117(16.93)		234(16.49)	
Do not want to discontinue medication because of ADR	108(14.84)		44(6.37)		152(10.71)	
I think it is meaningless to report ADR	65(8.93)		85(12.30)		150(10.57)	
unfilled	0(0.00)		3(0.43)		3(0.21)	
	*P* < 0.001*					

*Significant difference with *p* value < 0.05

## Discussion

To the best of our knowledge, this is the first KAP survey of ADR reporting for the general public in two cities with large differences in economic development in China. In this study, only about one-third of respondents had a good understanding of ADR reporting criteria. Our respondents’ average knowledge score was 6.49 out of a possible 10 points. Through the questionnaire survey, it can be said respondents’ knowledge of ADR and its monitoring and reporting was incomplete. Other studies have shown similar results [29, 35]. Studies [9, 39–41] show that the public (patients) and HCPs often ignore the criteria of reporting an ADR immediately upon suspecting an ADR, many individuals think that only serious ADRs that affect daily life or require hospitalization are worth reporting, or they only selectively report new ADRs, which will lead to underreporting. Only about half of respondents knew the definition of ADR, which was similar to the results of many studies [35, 42, 43]. Therefore, it is necessary to strengthen the public’s knowledge about ADRs and their monitoring and reporting. In statistical analysis, we found that differences in respondents’ gender, age, and education level all affect public awareness. This study showed that a higher proportion of older people have lower education levels. Most older adults have decreased liver and kidney function, weakened metabolic capacity, more comorbidities, and a large number of co-administered drugs, so they are at higher risk of ADRs [44, 45]. At the same time, people with lower education levels have a poor understanding of ADRs and ADR monitoring and reporting, limited ability to learn independently, and less access to health education and information [46, 47]. This suggests that relevant public education must be improved, using simple information that is understandable to older people and those with low educational levels.

In this study, respondents had positive attitudes about ADR reporting and monitoring. Most respondents believed that HCPs should inform the public (patients) about ADRs using detailed information. Many studies [48–52] have shown that HCPs are the main source of ADR-related knowledge for patients, while patients receive insufficient information about drug ADRs from HCPs, and many patients think that cooperation among HCPs is insufficient and cannot guarantee that they receive enough information about ADRs. This emphasizes the fact that medical workers must improve communication with the public by actively explaining to patients the importance of ADRs, monitoring, and reporting. HCPs should also strengthen cooperation and communication to better preserve patients’ safety.

Most people said they consult an HCP for information about ADRs before using a medication and feedback ADR information to HCPs, which was similar to other studies [6, 29, 42, 43]. Research has shown that when giving patients advice about medication, HCPs emphasize the purpose and manner of taking the medication but they tend to omit information about ADR monitoring and reporting [6]. Many patients believe that HCPs have not encouraged them to report ADRs [43]. As the main source of ADR-related information for the public, HCPs should provide ADR-related information to help patients cultivate safe medication practice and encourage them to report ADRs to a health professional in a timely manner, should an ADR occur. In this study, most respondents said that if there was a convenient way to report ADRs, they would take the initiative to do so. There are, however, currently no convenient channels for the public in China to report ADRs directly. The “Opinions on Strengthening the Adverse Drug Reaction Monitoring and Evaluation System and Capacity Building” issued by the China National Medical Products Administration [53] in July 2020 emphasized that in the future, China will enrich ADR reporting channels, explore the establishment of channels for patients/public to directly report ADRs. In the future, China should gradually establish a more complete and more convenient reporting channel through the promulgation of laws and regulations to facilitate the public to directly report ADRs. Our respondents mainly preferred reporting by telephone, as in other studies [25, 43, 54]. There were also more people who liked to report ADRs online. Other studies [6, 25, 46, 54] have also showed that some people have a tendency for reporting ADRs via SMS, email, completing a paper form, online, and other reporting methods. Thus, to improve the reporting rate and satisfy the needs and choices of different people, providing a variety of ADR reporting methods and channels is needed when establishing a public ADR reporting system. In this study, the main reasons respondents did not report ADRs were that they did not know the current channels by which to report and did not have a comprehensive understanding of ADR reporting criteria. ADR monitoring in China began relatively recently and there is little public information about ADR monitoring laws and regulations [55], which affects the public’s initiative to report ADRs. Many international studies [14, 25, 29, 43, 56] suggest other factors that may hinder public ADR reporting, such as insufficient understanding of ADRs and ADR monitoring and the reporting system, a lack of convenient reporting methods, a lack of follow-up after reporting an ADR, and a lack of feedback on the reported ADR, among others.

We found no significant differences in knowledge and attitude scores between respondents in the two cities in this study, but there were differences in respondents’ reasons for not reporting ADRs. More participants in Nanjing did not know that they should stop taking the drug when an ADR occurs and did not understand the importance of reporting an ADR. Therefore, greater attention is needed regarding these points in publicity and education for residents of Nanjing.

Most similar studies [6, 29, 43, 48, 50, 51] have rarely compared KAP of the general population in different regions. In this study, we selected two representative cities in eastern and western China with large differences in economic development in which to conduct our survey, to compare public KAP regarding ADRs and ADR monitoring and reporting. In this study, we found some differences in the educational level and economic status of participants surveyed in these two cities, but no significant differences in public knowledge and attitude scores between cities. One possible reason is that in this survey, we excluded respondents with a medical or pharmaceutical background. Therefore, residents of both cities without a medical background lacked extensive knowledge about ADR reporting and monitoring, which requires some professional expertise. This highlights that a lack of understanding about ADR monitoring and reporting is widespread among the general public and has less to do with regional economic development. Therefore, there is an urgent need for comprehensive and extensive education on ADRs, monitoring, and ADR reporting in all regions of China. China’s pharmacovigilance and related agencies should enhance information about the importance of ADRs and monitoring and reporting. At the same time, China must improve relevant laws and regulations and establish more complete ADR reporting channels for the public to directly report ADRs.

## Limitation

There are some limitations in this study. According to the China Statistical Yearbook 2019 [37], only 14.01% of the population had a college degree or above. In this study, the proportion of respondents with a college degree or above was far higher; for example, the proportion of respondents with a bachelor's degree was as high as 40.51%. This may be because Nanjing and Xi’an are capital cities of Jiangsu and Shaanxi provinces, respectively. Provincial capital cities are more economically developed and attract more people with higher education levels. At the same time, the proportion of respondents aged 18–30 in this study is relatively high, this may be because our research location is the provincial capital city. Therefore, the general knowledge of people in these cities toward ADR monitoring and reporting may be better and their attitudes more positive; thus, our study findings should be interpreted with caution. In order to ensure that the sample size is more representative, the corresponding statistical formula was used to calculate the sample size; and systematic sampling was performed according to the number of the report to avoid selection bias. The selected study sites are two provincial capitals in eastern and western China, as such, the sample size and sample representativeness have certain limitations. The knowledge, attitudes, and practice of the public in other provinces in eastern and western China needs to be further surveyed to provide a larger, more representative sample. As far as we know, no other research in China has compared the knowledge, attitudes, and practice of monitoring and reporting ADRs among the general public in two regions with large economic differences. Despite the above limitations, our study findings can enrich the research in this field and provide a reference point and basis for relevant policymakers to improve the ADR reporting system in China.

## Conclusion

In this study, we compare the knowledge, attitudes, and practice(KAP) regarding monitoring and reporting of ADRs among the general public in eastern and western China. There are no significant differences in knowledge and attitude scores between respondents in the two cities in this study, a lack of understanding about ADR monitoring and reporting is widespread among the general public and has less to do with regional economic development. Our study population had poor knowledge about ADR monitoring and reporting. There is an urgent need to conduct comprehensive education on ADR monitoring and reporting that is delivered to the public via multiple channels. As the main source of public access to ADR expertise, HCPs should allocate more time to discuss information about ADR monitoring and reporting with patients, and encourage them to actively report ADRs. Relevant departments can develop publicity and provide education via the Internet, news media, television, and other means to increase public awareness of drug safety and ADR reporting. China must explore ways to facilitate direct public reporting of ADRs by improving related laws and regulations.

## Supplementary Information

**Additional file 1.**

## Data Availability

All data generated or analysed during this study are included in this published article.
