# Supplementary material for: A cross-sectional study: comparison of public perceptions of adverse drug reaction reporting and monitoring in eastern and western China
Source: BMC Health Serv Res. 2022 Mar 8;22:318. doi: 10.1186/s12913-022-07720-0 (PMC8905784; doi:10.1186/s12913-022-07720-0)
Supplement: Supplementary file 1 — Additional file 1. [file 12913_2022_7720_MOESM1_ESM.docx]

**Questionnaire on "Knowledge-Attitude-Behavior" reported by the public on adverse drug reactions**

We are researchers in The Department of Pharmacy Administration and Clinical Pharmacy School of Pharmacy, Xi'an Jiaotong University. We would like to investigate the public's awareness of the reporting and monitoring of adverse drug reactions. We hope to get your support and cooperation. This survey is conducted anonymously, and the results are only used for scientific research.Please answer the questionnaire according to the actual situation.

Thank you for your great support!

School of Pharmacy Xi'an Jiaotong University

Ningsheng Wang; Bianling Feng

***Part 1*** ***Basic personal information***

1. Age: ____________

2. Nationality: ____________

3.Gender: A)Male B) Female

4.Educational level:

A) None/primary

B) Junior school

C) High school

D) Junior college

E) Undergraduate college

F) Master degree/above

5. Annual income level：

A) Under ¥30000

B) ¥30000-¥60000

C) ¥60000-¥90000

D) ¥90000-¥120000

E) ¥120000-¥150000

F) Above ¥150000

6. Are you currently engaged in a medicine-related occupation：

A) Yes A) No

7. Do you have a medicine-related education background：

A) Yes A) No

***Part 2. Respondents’ knowledge regarding ADRs***

**Do you think the following statement is True or False?**

( ) 1. Adverse drug reactions(ADR) are caused by the patient's use of substandard drugs, fake and inferior drugs, or overdose drugs.

( ) 2. After using drugs that have been on the market and are of qualified quality, if an adverse reaction occurs, it is a "medical accident".

( ) 3.The responsibility of the "drug safety monitoring department" is to collect information on ADRs that occur after the patient has taken the drug, and sort and analyze it.

( ) 4.For the drugs whose ADRs has been specified, the "drug safety monitoring department" does not monitor them.

( ) 5. "Drug safety monitoring related departments" only monitor serious ADRs (such as hospitalization or death of patients), and do not monitor mild ADRs.

( ) 6. According to national regulations, healthcare professionals such as doctors, pharmacists, and nurses can independently report information on ADRs.

( ) 7. According to national regulations, consumers (patients) can independently report ADR information.

( ) 8. Only when it is determined that the adverse reaction is caused by taking the drug, can the information on the ADR be reported.

( ) 9. After an ADR occurs, it can be reported within three months, and the report will not be allowed more than three months.

( ) 10. This city has established an ADR monitoring agency.

***Part 3. Respondents’ attitudes regarding ADRs***

**Do you agree with the following statement ?(single choice)**

1. Collecting adverse drug reactions can improve the safety of the drug.

A) strongly B) agree agree C) unsure D) disagree E) strongly disagree

2. Collecting information on ADRs is a benefit to the people, and each of us will benefit from it.

A) strongly B) agree agree C) unsure D) disagree E) strongly disagree

3. It is necessary for doctors and pharmacists to inform patients of ADR information in detail.

A) strongly B) agree agree C) unsure D) disagree E) strongly disagree

4. China should compel medical and healthcare personnel to report ADRs of patients.

A) strongly B) agree agree C) unsure D) disagree E) strongly disagree

5. China should formulate relevant policies to facilitate patients to directly report ADRs that occur during their medication.

A) strongly B) agree agree C) unsure D) disagree E) strongly disagree

6. If the "statutory ADR reporting personnel" fails to report the ADR of the patient, country should formulate relevant laws to punish them.

A) strongly B) agree agree C) unsure D) disagree E) strongly disagree

7. In order to improve the ability of medical staff to report ADRs, hospitals should conduct regular training.

A) strongly B) agree agree C) unsure D) disagree E) strongly disagree

8. Relevant departments should hold lectures to publicize the importance of ADR reporting to the public.

A) strongly B) agree agree C) unsure D) disagree E) strongly disagree

***Part 4. Respondents’ Practices regarding ADRs***

**(Q1-Q6:single choice; Q7:multiple choice）**

1.Would you consult doctors or pharmacists for ADR information while purchasing drugs？

A) Yes, I would.

B) No, I wouldn’t.

2. Would you check the “adverse drug reactions” section of the drug instructions？

A) Every time.

B) Most time.

C) Sometimes.

D) Never.

3. Do you suspect that ADR is occurring when you feeling sick？

A) Suspect and check the instructions for more information.

B) Suspect but not check the instructions for more information.

C) Not suspect.

4. What measures would you take when you have an adverse drug reaction？

A) Feed the information back to the medical staff.

B) Nothing was done.

C) Report to Pharmaceutical trading enterprises (drug store) .

D) Report to ADR monitoring center.

E) Report to pharmaceutical Manufacturing companies.

F) Exposure to the News Media.

5. If there is a policy that makes it easier for patients to report adverse drug reactions, would you take the initiative to report？

A) Yes, I would.

B) No, I wouldn’t.

6. Which way do you prefer to report adverse drug reactions?

A) By telephone.

B) By internet.

C) By email.

D) By post.

7. Why do you think you did not report adverse drug reactions? (multiple choices)

A) Do not know where to feedback ADR information.

B) ADR is not too serious to report.

C) Think it's too much trouble to report the ADR.

D) Do not want to discontinue medication because of ADR.

E) I think it is meaningless to report ADR.
